# Mussel-inspired Functionalization of Cotton for Nano-catalyst Support and Its Application in a Fixed-bed System with High Performance

**DOI:** 10.1038/srep21904

**Published:** 2016-02-23

**Authors:** Jiangbo Xi, Junwu Xiao, Fei Xiao, Yunxia Jin, Yue Dong, Feng Jing, Shuai Wang

**Affiliations:** 1 School of Chemistry and Chemical Engineering, Huazhong University of Science and Technology, Wuhan, 430074, China; 2 School of Chemistry and Environmental Engineering, Wuhan Institute of Technology, Wuhan, 430073, China; 3 School of Electrical and Information Technology, Yunnan University of Nationalities, Kunming 650031, China; 4 School of Physics, Huazhong University of Science and Technology, Wuhan, 430074, China

## Abstract

Inspired by the composition of adhesive and reductive proteins secreted by marine mussels, polydopamine (PDA) was used to coat cotton microfiber (CMF), and then acted as reducing agent for the growth of Pd nanoparticles on PDA coated CMF (PDA@CMF) composites. The resultant CMF@PDA/Pd composites were then packed in a column for the further use in fixed-bed system. For the catalysis of the reduction of 4-nitrophenol, the flow rate of the 4-aminophenol solution (0.5 mM) was as high as 60 mL/min. The obtained fixed-bed system even exhibited superior performance to conventional batch reaction process because it greatly facilitated the efficiency of the catalytic fibers. Consequently, its turnover frequency (TOF) was up to 1.587 min^−1^, while the TOF in the conventional batch reaction was 0.643 min^−1^. The catalytic fibers also showed good recyclability, which can be recycled for nine successive cycles without a loss of activity. Furthermore, the catalytic system based on CMF@PDA/Pd can also be applied for Suzuki coupling reaction with the iodobenzene conversion up to 96.7%. The strategy to prepare CMF@PDA/Pd catalytic fixed bed was simple, economical and scalable, which can also be applied for coating different microfibers and loading other noble metal nanoparticles, was amenable for automated industrial processes.

During the past decades, noble metal nanoparticles (NPs) have attracted considerable research interest in both fundamental studies and various practical applications because of their unique catalytic properties. Noble metal NPs can be directly employed as colloids in catalytic reactions^1^,^2^. Being ultrafine, they also offer many advantages over conventional bulk catalysts. Metal nanostructures possess a large surface area and abundant exposed low coordination sites, both of which are critical for their prominent catalytic activities^3^. However, the high surface energy of noble metal NPs makes them easily agglomerated or shape-changed during catalytic reactions, which results in the dramatic decrease of their activity and selectivity^4^,^5^,^6^. Besides catalysis, the separation, recovery, and recycling of catalyst are significant for achieving both economic and environmental advantages. However, the colloidal NPs are very difficult to be separated, often leading to serious aggregation during their separation and purification procedure. Thus, maintaining the catalytic activity and recycling of catalyst is highly desired^7^,^8^, especially in industrial applications.

Recyclability can be improved by anchoring NPs on a support material^9^,^10^,^11^. Various metal-support catalysts were prepared by depositing metal colloids on support materials, which not only extended their application areas but also improved their performances and recyclability^12^,^13^. However, few of them are amenable for automated process. Ideally for industrial applications, catalysts are usually loaded in an immobilized support (fixed bed)^14^. Numerous efforts have been made to construct a continuous flow reactor system^15^,^16^,^17^. However, both the flow rate and the catalytic activity were not satisfied. Chen’s group grew a dense Au nanowire forest on glass fibers, which enabled to use a porous support structure for an improved flow rate^18^. Thus, developing a catalytic fixed-bed system with high flow rate, high activity, and maximum recyclability is still a challenge. Moreover, the critical task remains to develop a facile strategy capable of directly anchoring various NPs onto fixed-bed materials in an economically feasible way.

As a natural product, cellulose offer many attractive properties, such as eco-friendliness, softness, degradability, availability, and low price. Thus, cellulose substances, such as paper, cloth, and crystalline cellulose nanofibers are ideal candidates as support for the immobilization of metal NPs. Therefore, many efforts have been devoted to immobilizing noble metal NPs on paper^19^,^20^, cloth^21^ and crystalline cellulose nanofibers^22^. However, they are not suitable for fixed-bed catalyst because of their sheet-like structure or nanosize. Instead of them, cotton microfibers (CMFs) are potential raw materials for fixed beds due to their excellent properties including microsize (centimeters in length), fibrousness and flexibility. To the best of our knowledge, the immobilization of NPs on raw CMFs has not been reported previously. Polydopamine (PDA), which is a mimic of the specialized adhesive foot protein, Mefp-5 (Mytilus edulis foot protein-5) secreted from mussels that developed via oxidative polymerization of dopamine (DA), can modify almost all material surfaces^23^,^24^,^25^,^26^. Furthermore, previous findings demonstrate that catechol groups contained in PDA were able to release electrons when oxidized into the corresponding quinone group, which triggered reduction processes of metallic cations^27^,^28^,^29^. Thus, PDA can be employed as a reducing agent to prepare PDA@NPs composites via direct redox reaction with metal salt^30^,^31^,^32^. In this regard, the adhesion of mussel protein might offer an alternative strategy for immobilizing noble metal NPs on CMFs under mild conditions.

Inspired by the unique properties (reductive and adhesive abilities) of DA secreted from mussels, herein, we utilized PDA to coat CMFs to prepare CMF@PDA composites firstly. Then, the PDA layer served as both reducing agent of metal ions and anchor for the resultant metal NPs on the surfaces of CMF@PDA (Fig. 1). After that, the resultant CMF@PDA/Pd composites were packed in columns to construct fixed-bed systems. We used a model reaction to evaluate the catalytic performance of these catalytic systems, in which 4-nitrophenol (4-NP) was reduced to 4-aminophenol (4-AP). The flow rate of the resulting 4-AP solution was up to 60 mL/min with a turnover frequency (TOF, TOF is defined here as the amount of 4-NP that 1 mmol Pd can convert into 4-AP per min) of 1.587 min^−1^ (see the video in the Supplementary Information). Furthermore, the catalytic system also exhibited excellent recyclability. The catalytic fibers could be recycled for nine successive cycles without a loss of activity.

## Results

**Figure 2:**
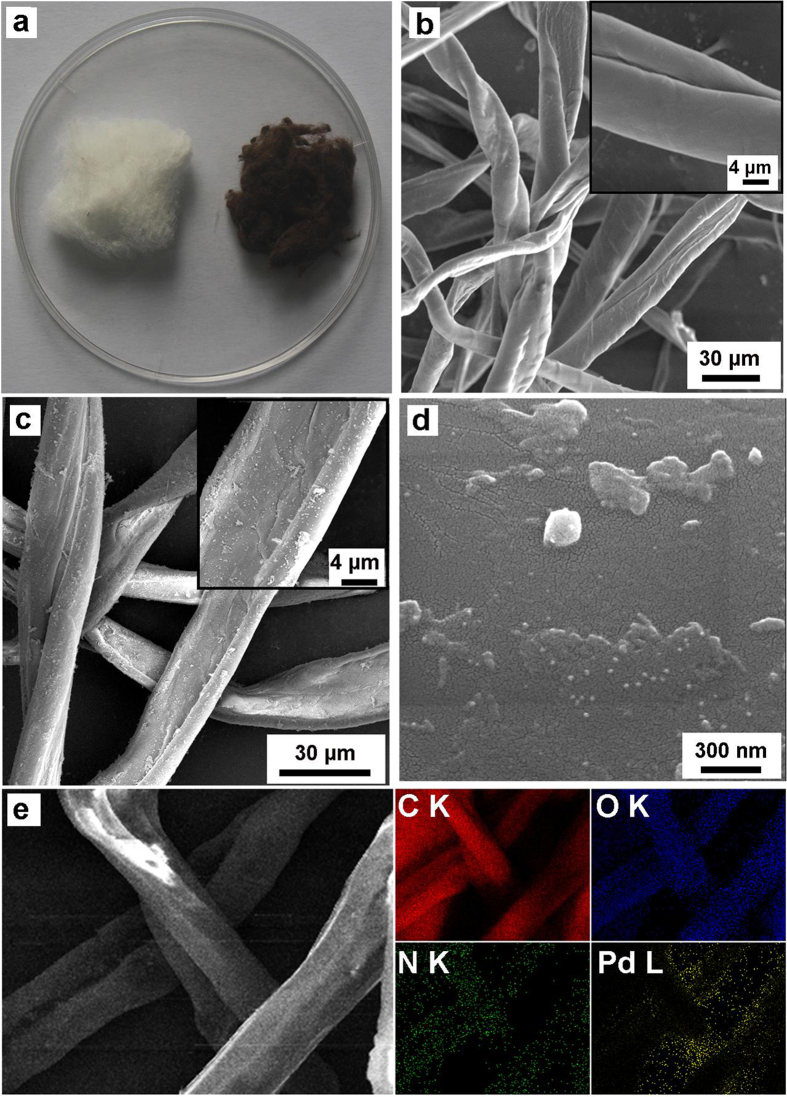
(**a**) Photograph of the cotton (left) and cotton coated by PDA (right). (**b**) SEM images of CMF. (**c**) SEM images of CMF@PDA/Pd. (**d**) High-magnification SEM image of CMF@PDA/Pd. (**e**) SEM mapping of CMF@PDA/Pd.

CMF@PDA was prepared by the self-polymerization of DA hydrochloride on the surfaces of CMF at a weak alkaline pH 8.5 (10 mM Tris buffer). Catechols tended to react with the hydroxyl groups, which result in the dehydration and formation of a charge-transfer complex. Then, hydroxyl-containing cellulose was coated with aqueous DA, and assemble layer-by-layer to form a PDA cladding layer on the surface of CMF eventually^26^. During the self-polymerization procedure, the change in color from white to brown was observed (Fig. 2a). The successful coating of CMF by PDA was also verified by scanning electron microscope (SEM) (Fig. 2b,c and inserts). After coating PDA, the surface of CMF became rough. Pd NPs were then grown onto the surface of CMF@PDA by immersing CMF@PDA in K_2_PdCl_4_ solution at 0 °C for 30 min. In this process, the PDA layer served as both the reducing and capping agent. In this regard, neither additional reductants nor metallic seed particles are needed. Pd NPs with an average diameter of ~14 nm were deposited on the surface of CMF@PDA, which was confirmed by counting ca. 50 Pd NPs from SEM image (Fig. 2d and Figure S1). The metal bonds at the N-sites and O-sites in PDA acted as the seed precursors for the formation of metal NPs with the continuous reduction of metal ions. Furthermore, the PDA coating plays an important role in preventing the metal NPs from agglomeration by the quinones and unoxidized catechol groups^22^. In particular, energy-dispersive X-ray spectroscopic (EDS) elemental maps of C, O, N, and Pd (Fig. 2e) clearly verified the homogeneous coating of PDA and the loading of Pd NPs on the surface of CMF@PDA support.

**Figure 3:**
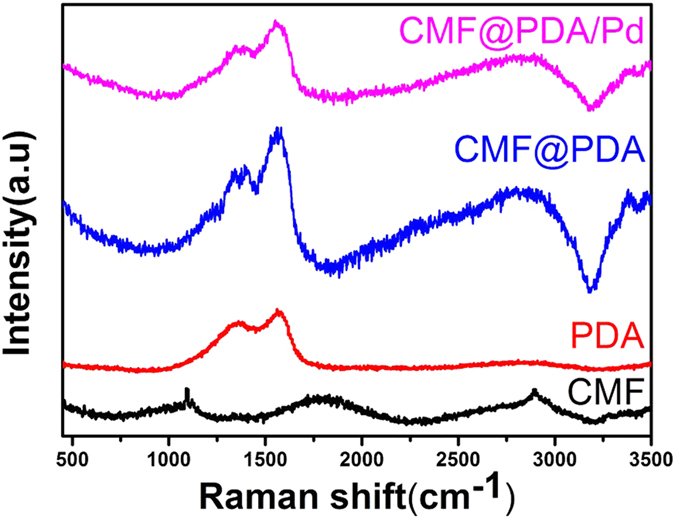
Raman spectra of CMF, PDA, CMF@PDA, and CMF@PDA/Pd composite.

**Figure 4:**
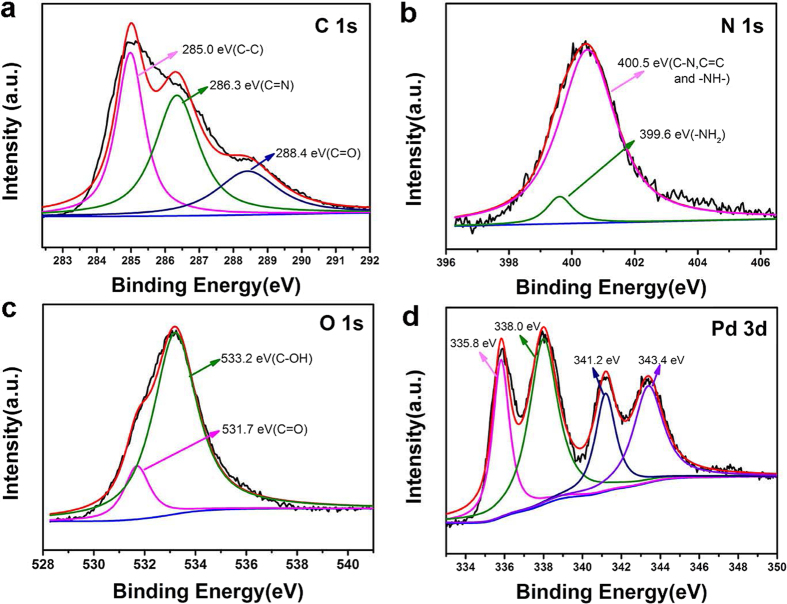
XPS spectra of the CMF@PDA/Pd composite. (**a**) C 1s, (**b**) N 1s, (**c**) O 1s, (**d**) Pd 3d.

To further investigate the surface composition, the catalytic fibers were also characterized by Raman spectroscopy, X-ray photoelectron spectroscopy (XPS), and microwave plasma-atom emission spectrometer (MP-AES). In Raman spectra, PDA exhibits broad peaks at 1580 and 1350 cm^−1^, resulting from stretching and deformation of aromatic rings, respectively (Fig. 3)^33^. CMF@PDA and the obtained CMF@PDA/Pd show mainly intense absorption features of PDA, indicating the successful wrapping of CMF by PDA layer. The XPS survey spectrum reveals the presence of C, N, O, and Pd signals in the sample (Figure S2), which were in agreement with the element compositions of PDA and Pd in the CMF@PDA/Pd compostite. The C 1s spectrum in Fig. 4a shows three components of C-C (285.0 eV), C = N (286.3 eV) and quinone C = O (288.4 eV), which came from the PDA layer. In the N 1s spectrum (Fig. 4b), the peak located at 399.6 eV was assigned to the component of −NH_2_^34^, the broad peak at 400.5 eV was attributable to C-N, C = N- and -NH-35. Obviously, these nitrogen-containing groups originated from PDA. Additionally, the O 1s spectrum in Fig. 4c shows the oxygen signals of quinone C = O (531.7 eV) and phenolic hydroxyl C-OH (533.2 eV) originating from the PDA layer of CMF@PDA/Pd composite^34^. Furthermore, there are two spin-orbital doublets (Fig. 4d), which are accounted for by two electronic states of palladium, i.e., Pd(0) (binding energy 335.8 and 341.2 eV) and Pd(II) (338.0 and 343.4 eV)^36^, indicating the successful loading of Pd NPs in the CMF@PDA/Pd composite. The Pd(II) may attribute to the adsorbed K_2_PdCl_4_ by CMF@PDA composite. The Pd content in the CMF@PDA/Pd composite was as low as 0.409 wt.%, which was determined using MP-AES.

**Figure 5:**
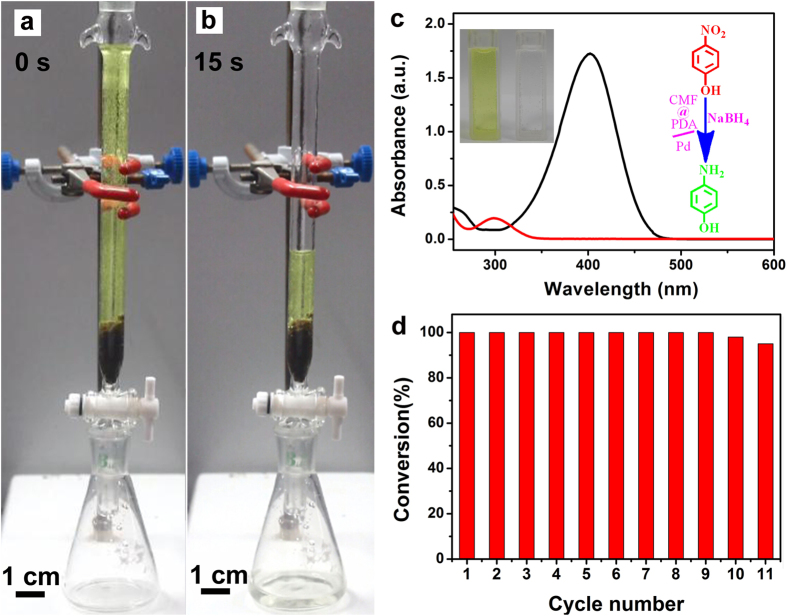
(**a,b**) Photographs showing the setup and the complete reduction of 10 mL of 4-NP (0.5 mM) in 15s. (**c**) UV/Vis spectra of aqueous mixture of 4-NP and NaBH_4_ before (black) and after (red) the reaction solution was allowed to flow through the catalytic fixed-bed system, the color changes of the conversion (the left insert), reaction scheme of the catalysis (the right insert) and (**d**) Graphic illustration of the conversion of 4-NP in 20s versus the number of catalytic cycles.

**Figure 6:**
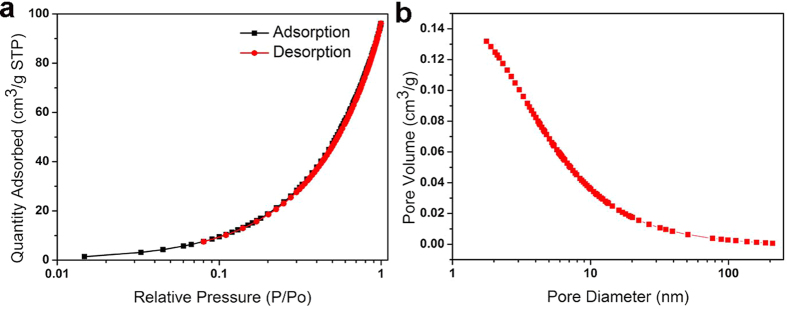
(**a**) N_2_ adsorption/desorption isotherms at 77 K and (**b**) the pore size distribution diagram of CMF@PDA/Pd composite.

To evaluate the performance of the fixed-bed system, we used a model reaction in which 4-NP was reduced to 4-AP using NaBH_4_ as the reductant. Briefly, we packed a few pieces of catalytic fiber (492 mg with 0.409 wt.% Pd) in a column (d = 1 cm) to achieve about 3 cm in height. Aqueous mixture of 4-NP (0.5 mM) and NaBH_4_ (0.3 M) were then added into the column. During the catalytic process, the complete reduction of 4-NP to 4-AP was detected by color change as well as the UV-vis spectra^37^. As shown in Fig. 5a,b and the video in Supplementary Information, the reaction mixture remained bright yellow until it was allowed to flow through the catalyst fixed-bed. After flowing through the catalytic system, the exiting solution was completely colorless, indicating the full reduction of 4-NP. It had been reported that the aqueous mixture of 4-NP and NaBH_4_, and aqueous 4-AP had maximum absorption at 400 and 295 nm, respectively^38^. As shown in the UV-vis absorption (Fig. 5c), the absorption peak of 4-NP completely disappeared after the reaction solution was allowed to flow through the catalytic fixed-bed system; Meanwhile, the absorption peak of 4-AP appeared accordingly. The catalytic fixed-bed system showed a TOF as high as 1.594 min^−1^, which may attribute to the moderate specific surface area (156.5 m^2^/g) and mesoporous structure of catalytic fibers (Fig. 6).

As an important parameter, the flow rate of the catalytic fixed-bed system was also investigated. In the general case, our catalytic column (d = 1 cm, h = 3 cm) can only achieve a flow rate of 5 mL/min. External pressure can be applied to the catalytic fixed-bed system to improve its flow rate (see the video in the Supplementary Information). Typically, it could take only 20s to process 20 mL of 0.5 mM 4-NP under pressure with a TOF of 1.587 min^−1^. As a result, the flow rate is up to 60 mL/min, which is higher than that of some recently reported catalyst (Table 1). The recyclability and stability of catalytic fiber were investigated by repeating the reduction reaction (Fig. 5d). The catalytic fibers could be recycled for nine successive cycles without a loss of activity. The conversion was up to 95% even in the 11^th^ reaction cycle. As shown in Figure S3a, the used catalytic fibers show no color change comparing with the freshly-made ones, and the PDA layer of recycled CMF@PDA/Pd composite was steadily coated on the surface of CMF (Figure S3b). It is reasonable to conclude that Pd NPs were still anchored on the surface of CMF@PDA after repeated reaction cycles. More importantly, this synthesis route is a versatile and can also be applied for coating different microfibers (Figure S4) and loading other noble metal nanoparticles, such as Au, Pt, and Ag.

Taking into account that the Suzuki coupling reaction is a widely utilized reaction for aryl-aryl bond formation in the synthesis of biaryl compounds, such as pharmaceuticals, herbicides and natural products, as well as engineering materials^40^, we further chose the Suzuki coupling reaction as a model reaction to evaluate the performance of the fixed-bed system. Our results showed that the reaction solution could be catalyzed by the catalytic fibers when flowed through the fixed-bed, and the conversion of iodobenzene was as high as 96.7% (Figure S5).

## Discussion

To elucidate the excellent catalytic performance of our fixed-bed system, we also performed a conventional catalytic test as a comparison. Briefly, 4-NP (3 mL, 0.1 mM) was mixed with aqueous solution of NaBH_4_ (0.1 mL, 0.3 mM). Then, catalytic fibers (10 mg) were added with constant magnetic stirring. The reduction of 4-NP catalyzed by those CMF@PDA/Pd composites was finished within 1.2 min with a TOF of 0.643 min^−1^. The efficiency of the fixed-bed system was two times higher than the conventional batch reaction process, indicating that this fixed-bed system could greatly facilitate the activity of the catalytic fibers. It is believed that the super performance of the fixed-bed system was attribute to the excellent mass transportations, which make the reactants contact effectively with the Pd NPs anchored on the surface of CMF@PDA. In conventional catalytic process, the CMF@PDA/Pd catalytic fibers aggretated into a bulk cluster after stiring, which was difficult to isolate individual cellulose fibrils (Figure S6). As a result, the mass transportations between catalytic fibers and reactants were inhibited and eventually limited the catalytic performance. Furthermore, it is believed that the PDA coating could change the surface property of CMF from non-wettable to wettable, resulting in enhanced accessibility to the reactants. The wettability measurement showed that water droplets can easily infiltrate into the water-absorptive fibrous networks after the surface modification of cottons with PDA (Figure S7). This may attribute to the waxes and fatty substances contained in natrual cotton, which decrease its wettability. For comparison, CMF/Pd composite with similar (Pd conten 0.447 wt.%) was also examined for the catalytic reaction and exhibited relatively low activity (TOF = 0.859 min^−1^), possibly due to their poor wettability, low mass transfer of the support (cotton)^41^,^42^. Therefore, 4-NP aqueous solvent can flow through the CMF@PDA/Pd catalytic fibers rapidly and achieve effective contact with the ultrafine Pd NPs anchored by PDA, which leads to the high flow rate and TOF in this fixed-bed system. As discussed, the presence of the PDA coating was efficient as a capping agent to stabilize the nanoparticles on the cotton surfaces. Therefore, the catalytic fiber demonstrated excellent recyclability and stability.

In conclusion, PDA-coated CMFs have been easily prepared through one-step self-polymerization of dopamine hydrochloride on CMFs bulk at room temperature. Pd NPs can be deposited and stabilized on CMF@PDA via spontaneous reduction between K_2_PdCl_4_ and reductive PDA coating and followed by reduction of NaBH_4_. The fixed-bed systems constructed by packing resultant CMF@PDA/Pd composites into columns exhibited high activity, flow rate, and recyclability in the reduction reaction of 4-NP. Their efficiency is even higher than conventional batch reaction process, indicating that the fixed-bed system greatly facilitated the activity of the catalytic fibers. Furthermore, the catalytic system based on CMF@PDA/Pd can also be applied for Suzuki coupling reaction with the iodobenzene conversion of up to 96.7%. The strategy to prepare CMF@PDA/Pd catalytic fixed-bed is simple, economical, and scalable. This synthesis route can also be applied for coating different microfibers and loading other noble metal nanoparticles. This system supplies a platform to fabricate catalytic fixed bed, which can be employed in a broad range of noble metal catalyzed reactions. We envision that the special CMF@PDA/Pd composite will have broad applications in many fields including environment and material science.

## Methods

### Synthesis of CMF@PDA

The bulk cotton cluster should be teared into small pieces before use because the fibres are prone to aggregate as a result of hydrogen bonds from hydroxyl groups in cellulose. Typically, 300 mg natural cotton was immersed in 70 mL 3 mg/mL DA hydrochloride Tris solution (pH 8.5, 10 mM Tris buffer) and allowed to proceed for 48 h under stirring at room temperature. The resultant product was separated and collected, and subsequently put through five wash cycles and dried by freeze drying.

### Synthesis of CMF@PDA/Pd

250 mg CMF@PDA was immersed in a 50 mL K_2_PdCl_4_ (10 mM) aqueous solution. The mixture was stirred at 0 °C for 0.5 h. After the reaction, the resulting Pd NPs-deposited CMF@PDA was separated from the suspension, and subsequently washed with ultrapure water five times. After that, the resultant CMF@PDA/Pd composites were subsequently immersed into an aqueous NaBH_4_ solution for 30 min to reduce the adsorbed K_2_PdCl_4_. Finally, the obtained CMF@PDA/Pd was washed by ultrapure water for five times and dried by freeze drying.

### Synthesis of CMF/Pd

2.50 g CMF immersed in a 200 mL K_2_PdCl_4_ (1.80 mM) aqueous solution for 10 min. Then 80 mg of NaBH_4_ was add to the mixture and stirred at 10 °C for 0.5 h to reduce the K_2_PdCl_4_. After the reaction, the resulting Pd NPs-deposited CMF was separated from the suspension. Finally, the obtained CMF/Pd composite was washed by ultrapure water for five times and dried by freeze drying.

### Catalytic performance of the fixed-bed system

492 mg the catalytic fibers were packed in a column (d = 1 cm) to reach about 3 cm in height. Then, 20 mL aqueous mixture of 4-NP (0.5 mM) and NaBH_4_ (0.3 M) was added into the column. UV/Vis absorption spectra were recorded to monitor the change in the reaction mixture.

### Catalytic performance of the conventional batch reaction process

3 mL aqueous 4-NP solution (0.1 mM) was mixed with 0.1 mL of aqueous NaBH_4_ solution (0.3 mM) in a 5 ml vial. Then, 10 mg of catalytic fibres were added with constant magnetic stirring at a stirring speed of 600 rpm. UV/Vis absorption spectra were recorded to monitor the change in the reaction mixture.

### Catalytic performance of the fixed-bed system for Suzuki coupling reaction

4.0 g the catalytic fibers were packed in a column (d = 1 cm). Then, the fixed-bed system was kept in 80 °C oven. After that, 20 mL ethanol solution containing iodobenzene (0.25 mM), benylboronic acid (0.5 mM) and K_2_CO_3_ (1 mM) was preheated to 80 °C, and subsequently added into the column. 20 mL mixture could flow through the fixed-bed within 20 min, and the exiting solution was then analysed by high-performance liquid chromatography (HPLC).

## Additional Information

**How to cite this article**: Xi, J. *et al.* Mussel-inspired Functionalization of Cotton for Nano-catalyst Support and Its Application in a Fixed-bed System with High Performance. *Sci. Rep.* **6**, 21904; doi: 10.1038/srep21904 (2016).

## Supplementary Material

Supplementary Information

Supplementary Video

## Figures and Tables

**Figure 1:**
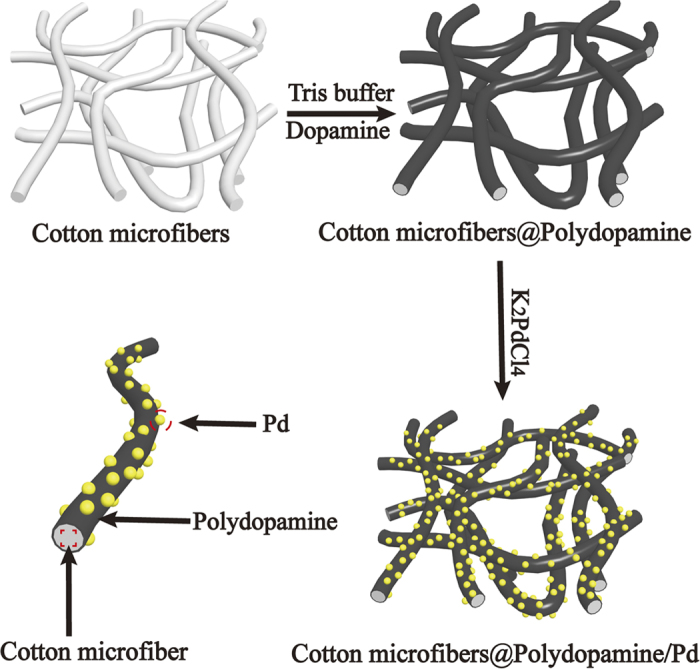
Schematics of formation of CMF@PDA/Pd composite.

**Table 1:** Comparison of the flow rate and TOF in the reduction of 4-NP as reported in the literature.

Catalysts	Metal content (wt.%)	Flow rate (mL/min)	TOF (min^−1^)	Ref.
CMF@PDA/Pd	0.409	60	1.587	This work
Glass fiber-AuNW forest	2.2	32	5.40	18
Silica spheres with AuNPs	NA	1.2	1.80	18
Anodic alumina-AuNPs membrane	NA	17.4	NA	39
Pd-rGO-CNT	1.12	NA	1.17	10
Ag@C nanofiber	8.4	NA	0.58	6
