# Supplementary material for: Mussel-inspired Functionalization of Cotton for Nano-catalyst Support and Its Application in a Fixed-bed System with High Performance
Source: Sci Rep. 2016 Feb 23;6:21904. doi: 10.1038/srep21904 (PMC4763215; doi:10.1038/srep21904)
Supplement: Supplementary Information [file srep21904-s1.doc]

**SUPPLEMENTARY INFORMATION**

**Mussel-inspired Functionalization of Cotton for Nano-catalyst Support and Its Application in a Fixed-bed System with High Performance**

Jiangbo Xi^1,2^, Junwu Xiao^1^, Fei Xiao^1^, Yunxia Jin^3^, Yue Dong^1^, Feng Jing^4^ and Shuai Wang^1^

**Materials.** All solutions were prepared using deionized water (resistivity > 18 MΩ·cm^-1^).Cotton microfiber was purchased from Xinshenshi Chemical Reagent Co. (China).K_2_PdCl_4_ (99%)was purchased from Sinopharm Chemical Reagent Co. (China). Dopamine hydrochloride (98%) and tris (hydroxymethy1) aminomethane (Tris, 99.8%) were purchased from Aladdin Chemistry Co., Ltd. (China).

**Characterization**. The morphology and structure of products were characterized with a field-emission scanning electron microscope (SEM, FEI, Nova NanoSEM 450). The specific surface area was measured with Micromeritics ASAP2020. Surface Area and Porosity Analyzer were calculated using the Brunauer-Emmett-Teller (BET) equation. Raman spectrum was measured by a confocal laser micro-Raman spectrometer (DXR, USA) equipped with a He–Ne laser of excitation of 532 nm at a laser power of 0.6 mW. X-ray photoelectron spectroscopy (XPS) measurements were performed on VG ESCALAB 250 spectrometer with monochromatic Al Kα (1486.71 eV) X-ray radiation (15 kV and 10 mA) and hemispherical electron energy analyzer. The UV-vis measurements were performed on a UV-2550 spectrophotometer (Shimadzu, Japan). The Pd contents in the catalysts were determined using microwave plasma-atom emission spectrometer (MP-AES, Agilent 4100, USA).Reagent conversions and product yield were determined by High-performance liquid chromatography (HPLC) analysis. HPLC analysis was performed on a Waters chromatograph (USA) equipped with a Zorbax Eclipse XDB-C18 4.6x150 mm column (Agilent, USA). The content of the reactants and product were calculated from the ratio of the peak area at the wavelength 254 nm．


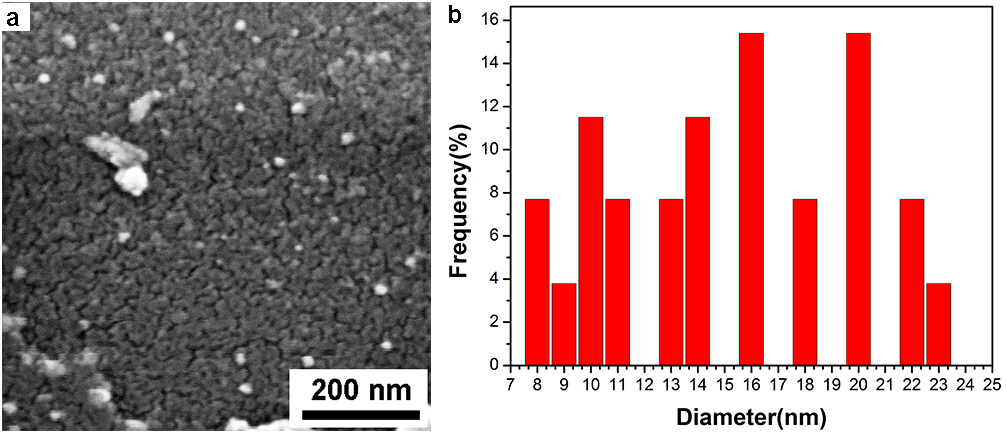


**Figure S1.** (a) High-magnification SEM image of CMF@PDA/Pd composite and (b) statistic histogram of the Pd particles size distribution on CMF@PDA/Pd composites.

**
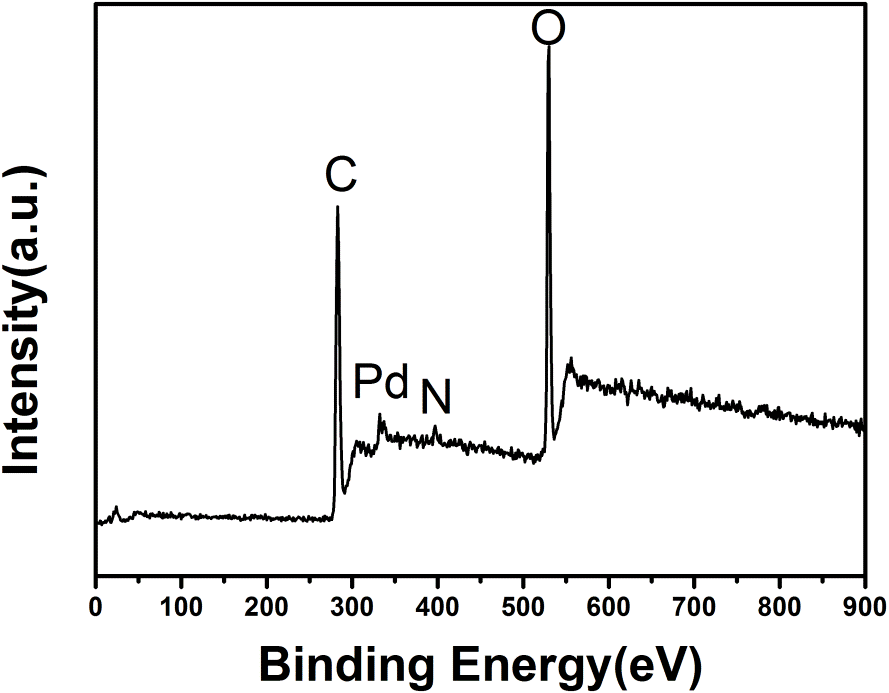
**

**Figure S2.** XPS survey spectra of CMF@PDA/Pd composite.

**
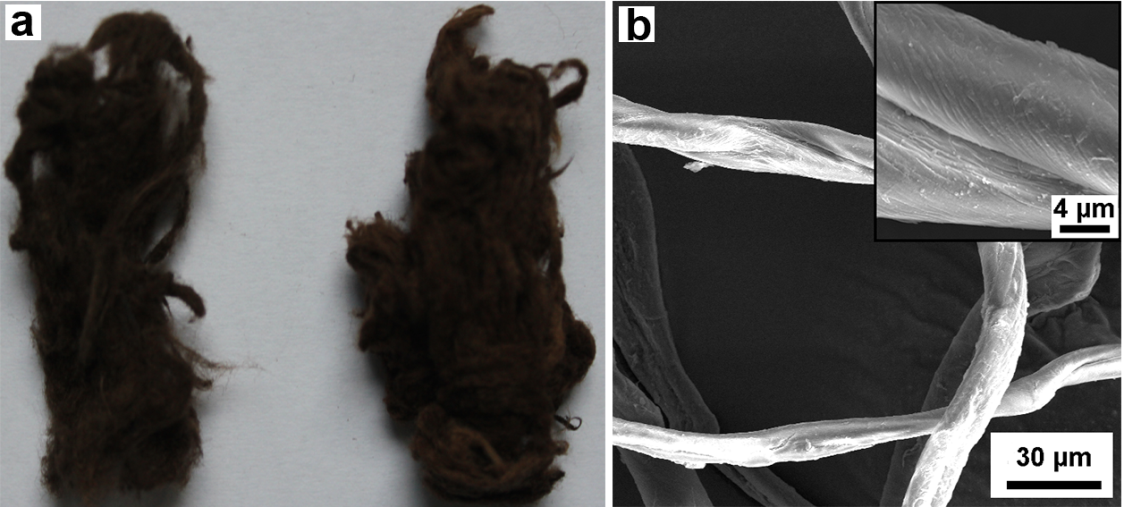
**

**Figure S3.** (a)Photograph of the CMF@PDA/Pd composite before (left) and after (right) reaction cycles, and (b) SEM images of CMF@PDA/Pd composite after nine cycles.

**
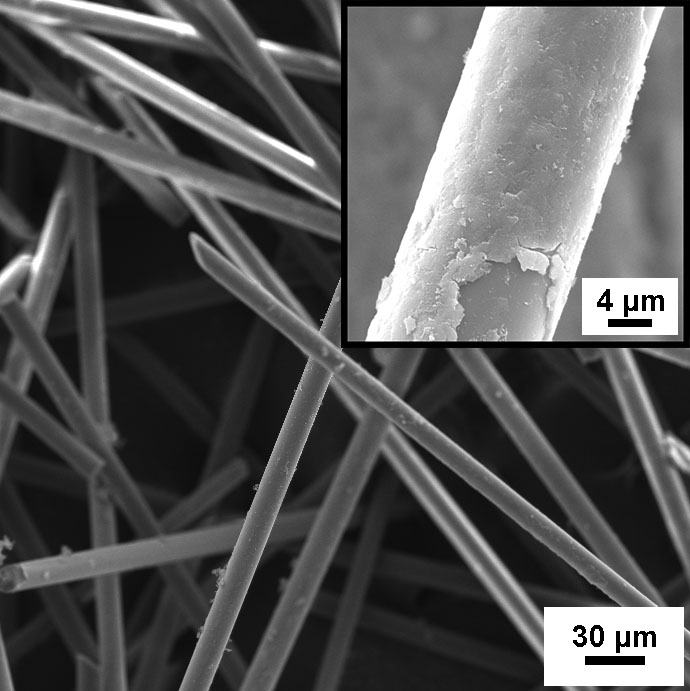
**

**Figure S4**. SEM images of glass microfiber@PDA/Pd composite.

**
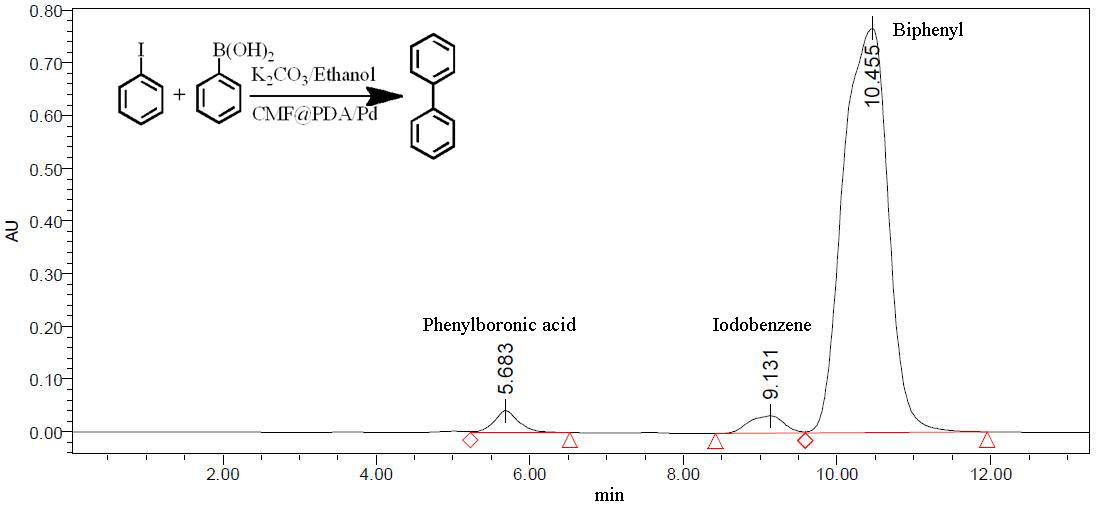
Figure S5.**Chromatogram of exiting solution of Suzuki reaction flowed through the fixed-bed system, the reaction scheme of Suzuki reaction (insert). The separation was carried out on a Zorbax Eclipse XDB-C18 column with 90% (v/v) ethanol in H2O; flow-rate at 0.5 mL/min; detection at 254 nm.


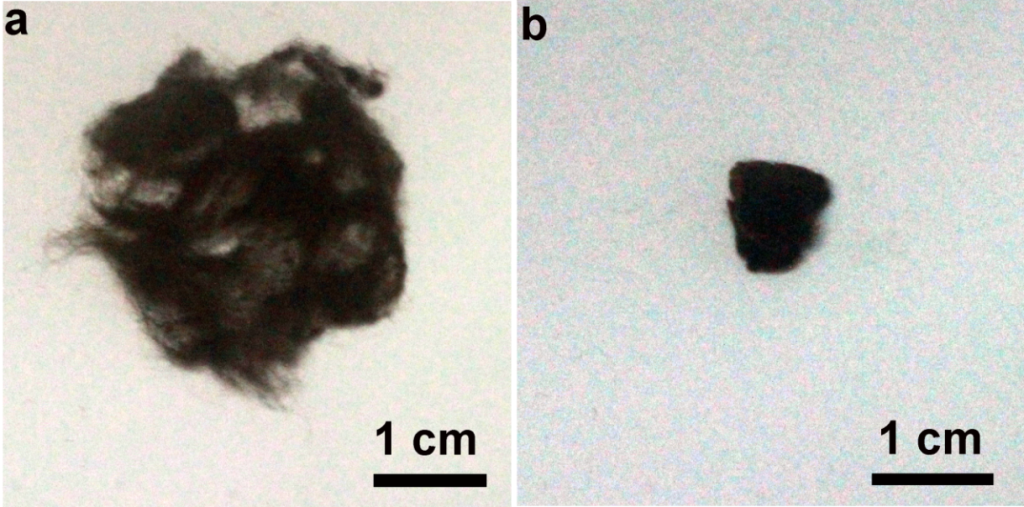


**Figure S6.** Photograph of the CMF@PDA/Pd composite before(a) and after(b)batch reaction process.

**
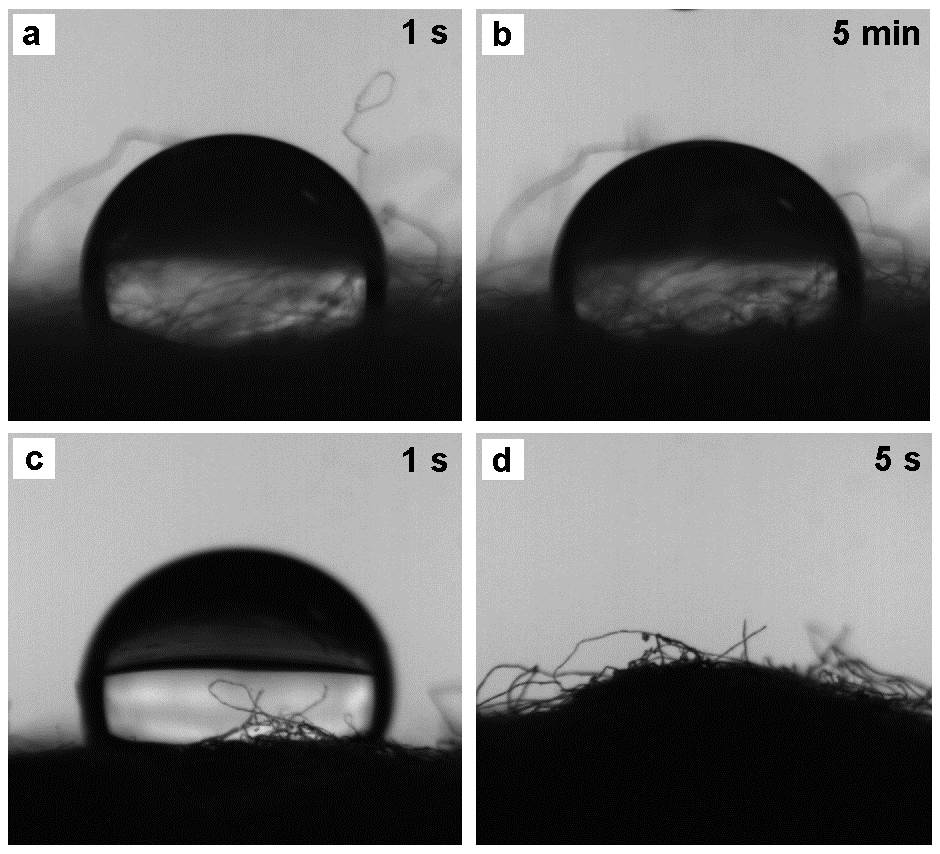
**

**Figure S7.** Pictures of water droplet on CMF (a, b) and CMF@PDA composite (c, d).

**Supplementary Information (Video).** 20 mL of 0.5 mM 4-NP can flow through the catalytic fixed-bed within 20 s. The reaction mixture remained bright yellow until it was allowed to flow through the catalytic fixed-bed. After flowing through the catalytic system, the exiting solution was completely colorless, indicating the full reduction of 4-NP.
